# Evaluation of Antimicrobial Activity, Cytotoxicity, and Phytochemical Composition of *Ocimum americanum* L. (Lamiaceae)

**DOI:** 10.1155/2022/6484578

**Published:** 2022-03-17

**Authors:** Hashim Ali, Joseph Nguta, Fredrick Musila, Isaac Ole-Mapenay, Dorine Matara, James Mailu

**Affiliations:** ^1^Department of Public Health, Pharmacology and Toxicology, Faculty of Veterinary Medicine, University of Nairobi, Nairobi, Kenya; ^2^Department of Applied and Technical Biology, School of Biological and Life Sciences, Technical University of Kenya, Nairobi, Kenya

## Abstract

**Background:**

Herbal plants are a natural source of novel biomolecules used widely in ethnomedicine. The present study was intended to examine the antimicrobial properties, cytotoxicity, and phytoconstituents of *Ocimum americanum* L., an herb traditionally used by the people of Swahili (Kenya) against microbial infections.

**Methods:**

The aerial parts of *Ocimum americanum* L. were sourced, dried, milled, and extracted using three solvents: aqueous, acetonic, and 70% hydroethanolic. Additionally, fractions of chloroform and ethyl acetate were obtained from all crude extracts of the plant. The antimicrobial property was evaluated using agar well diffusion and microdilution techniques against human opportunistic pathogens including *S. aureus*, *E. coli*, and *C. albicans*. The brine shrimp cytotoxicity test was used to analyze the lethality of the extracts and fractions. Phytochemical screening was used to qualitatively assay the presence of phytoconstituents.

**Results:**

The phytochemical assay confirmed the presence of alkaloids, phenols, flavonoids, tannins, saponins, terpenoids, reducing sugars, anthraquinones, and glycosides. The lethality test demonstrated that all the extracts and fractions were toxic against *Artemia salina* nauplii with LC_50_ values ranging from 0.59 to 559.71 *µ*g/ml. Chloroformic fraction of the hydroethanolic extract had the highest lethality with an LC_50_ value of 0.59 *µ*g/ml. Two of the extracts and their fractions displayed antimicrobial activity against the Gram-positive bacteria (*B. cereus* and *S. aureus*) and fungus (*C. albicans*), while the same extracts had no activity against the Gram-negative bacteria (*E. coli* and *K. pneumoniae*). The highest antimicrobial activity was seen in the ethyl acetate fraction of the hydroethanolic extract at 250 mg/ml against *Bacillus cereus* which had an inhibition zone of 26.00 ± 0.00 and MIC value of 62.5 mg/ml.

**Conclusion:**

In the current study, we report that *Ocimum americanum* L. demonstrated moderate antimicrobial activity, contains numerous phytocompounds, and is highly cytotoxic; thus, further research is needed for bioprospecting a novel compound.

## 1. Introduction

All the antimicrobial drugs that are introduced into the market have limited clinical use due to acquired or intrinsic mechanism of resistance of microbes [1]. Antimicrobial drug resistance (AMDR) has emerged due to the undiscerning utilization of antibiotic feed additives in animal husbandry and intensified by noncompliance of patients to the prescribed antimicrobial dosage regimens [1, 2]. Additionally, the reemergence of infectious diseases (e.g., smallpox and poliomyelitis) and the high cost of antimicrobials have been major factors contributing to futile management of communicable diseases in the third world countries such as Kenya [2, 3]. Consequently, alternative strategies are needed to fight antimicrobial resistance, and the plant kingdom serves as natural reservoir at our disposal [4]. Moreover, ethnopharmacology is a very valuable tool in research and development which leads to the discovery and development of novel bioactive compounds [5].

The medicinal plant *Ocimum americanum* L. is native to the tropics and subtropical parts of the world including Africa and Indian subcontinents. The herbal plant has a wide phytogeographical distribution in Eastern Africa, making the herb the most popular botanical in the region [6]. In Kenya, it is distributed extensively in the forest boundaries, grassland and secondary bushland, dry regions, and riverine sites, mostly in the knolls [7]. It is a small upright branched, sweet-smelling yearly perennial shrub which reaches up to 1 m in length. Stems are hairy, slightly rounded, and appressed near the base. Leaves are typically hairless ovate in shape and up to 25 mm in length [8].

Depending on the locality and differences of the dialect, the Swahili people of East Africa refer to the basil (*Ocimum*) as *Mrihani/Kivumbani/Mvumbani* [9]. *Ocimum americanum* L. is used for therapeutic and nontherapeutic purposes in the diverse ethnic groups in Africa [9, 10]. Essence from the herbs is used as body cologne, tobacco flavoring, and mint in tea. Basil leaves and branches are frequently used as insect repellents and insecticides against mosquitoes, bees, flies, and other insects. The leaves and/or branches are smoked or placed on the rooftop to give the repulsive effect. In addition, either the leaves can be crushed between the palms of the hands and inhaled, or hot aqueous vapor can be inhaled to clear anterior nares and used as therapy for bronchial phlegm [11, 12]. Concoctions from the plant are used for treating haemorrhoids (piles), coughs, tuberculosis (TB) stomachache, and eye and ear complaints [4]. Decoctions of the leaves are used to treat peptic ulcers and also as antipurging agents in East Africa. The Swahili people use aerial parts (decoction) of the herb for controlling hypertension, to cure stomach pain and as mint in tea [13]. In Tamil Nadu (India), it is commonly called *Nai thulasi*, and its decoction (leaf extract) is customarily used to treat diabetes, dysentery, haemorrhoids, diarrhoea, and constipation [4].

Lamiaceae, the mint family, is well known for its aromatic herbs of balms and mints. The genus *Ocimum* (basil) comprises more than 50 species of aromatic plants, some of which have excellent therapeutic activity with a potential for isolation of a new bioactive compound [14]. The main important property of the Lamiaceae (mint family) is attached to its content of essential oils. The essential (volatile) oils obtained from the mint herbs are a natural blend of bioactive compounds with robust fragrance, formed during secondary metabolism. Also termed as volatile oils due to their high unstable property, they are a rich mix with broad spectrum of biological properties [15]. *Ocimum americanum* L. has a wide range of ethnopharmacologically active compounds in the form of volatile oils. These include 1,8-cineole, linalool, methyl eugenol, eugenol, *trans*-caryophyllene, and various terpenenes [10, 16]. In addition, it is reported to have numerous biophysiological activities, including acute peptic ulcer as well as gastric cytoprotective antiulcer [15], antifungal [17], antimicrobial [4, 18], and larvicidal activity [19].

## 2. Materials and Methods

### 2.1. Chemicals, Reagents, and Drugs

Acetone (Loba Chemie Pvt. Ltd., India), analytical-grade ethanol (Loba Chemie Pvt. Ltd., India), chloroform (Sigma-Aldrich Pty Ltd., Germany), ethyl acetate (Loba Chemie Pvt. Ltd., India), *Artemia salina* eggs (Dohse Aquaristik GmbH & Co. KG, Germany), Mueller–Hinton agar (MHA) (HiMedia Laboratories Pvt. Ltd.), Mueller–Hinton Broth-MHB (HiMedia Laboratories Pvt. Ltd.), ceftriaxone (Makcur Laboratories Ltd.), terbinafine (Beximco Pharmaceuticals Ltd.), dimethyl sulfoxide-DMSO (Thomas Baker (Chemicals) Pvt. Ltd., India), and vincristine sulphate (Celon Laboratories Ltd.) were used in the current study; the reagents and chemicals were of analytical grade.

### 2.2. Plant Material Collection

The medicinal plant *Ocimum americanum* L. was collected from Pate Island, Lamu County (2.1090°S, 41.0600°E). Coast region, in July 2021 and transported to the Department of Public Health, Pharmacology and Toxicology, University of Nairobi. The plant authentication and identification were done at the East African Herbarium of the National Museums of Kenya by Mr. Mathias Mbale. The plant specimen was deposited, and voucher specimen reference number of NMK/BOT/CTX/5/1/3 was issued for the sample plant.

### 2.3. Extraction and Fractionation

The plant materials were washed with portable water, rinsed with purified water, and air-dried at room temperature to a constant weight. The dried plant materials were pulverized to an even powder by means of an electric mill. The pulverized material (500 g) was cold macerated in 1000 ml of the solvent, acetone, distilled water, and 70% hydroethanol, for 72 h at room temperature and stirred three times a day. The crude extracts were filtered using a Whatman filter paper No. 1. The aqueous crude extract was lyophilized by a freeze dryer to dry powder. The hydroethanolic crude extract was concentrated in a rotary evaporator and then lyophilized with a freeze dryer. The acetone crude extract was concentrated with rotary evaporator and then dried on a sand bath (40°C) till constant weight. The crude extracts were then vialed, labeled, and stored at 4°C, until use. Liquid-liquid fractionation (LLF) was done as described by Kuo et al. [20] method with modifications. The crude extracts obtained from acetone, aqueous, and hydroethanolic solvents were partitioned in chloroform and ethyl acetate using LLF. The extracting layer was concentrated with rotary evaporator at 40°C to remove the solvent, and the fraction was dried to constant weight on a sand bath at 40°C. The dried fractions of chloroform and ethyl acetate were refrigerated at 4°C before analysis.

### 2.4. Antimicrobial Assay

#### 2.4.1. Test Microorganisms

The microorganisms strains used in this research were from the American Type Cell Collection (ATCC) standard. They were four bacterial strains and one fungal strain. Gram-positive bacteria included *Staphylococcus aureus* (ATCC 25925) and *Bacillus cereus* (ATCC 11778) while Gram-negative included *Klebsiella pneumoniae* (ATCC 700603), *Escherichia coli* (ATCC 25922), and *Candida albicans* (ATCC 10231). They were provided by Microbiology Laboratory, Department of Public Health, Pharmacology and Toxicology, Faculty of Veterinary Medicine, University of Nairobi.

#### 2.4.2. Microbial Culture

The choice of test microbes was based on the recommendation of Clinical Laboratory Standards Institutes (CLSI) that standard test microorganism be used as clinical standard. The standard microbes were subcultured on a blood agar and maintained at 4°C till use. Additionally, a suspension of each microbial culture was prepared by suspending a loopful of the microbes on 10 ml sterile physiological saline to give a concentration of 0.5 McFarland. Furthermore, stock solution of 500 mg/ml was prepared by triturating 2 g of each extract and/or fraction in 1 ml of 1% DMSO and then mixed with 3 ml of sterilized media to make up to 4 ml. Twofold serial dilution of 250 mg/ml, 125 mg/ml, 62.5 mg/ml, 31.25 mg/ml, 15.625 mg/ml, and 7.8125 mg/ml was carried out from the stock solution.

#### 2.4.3. Agar Well Diffusion Technique

Agar well diffusion assay as described by Njue et al. was used to evaluate the zone of inhibition of the three crude extracts and six fractions of *Ocimum americanum* L. [21]. The MIC (minimum inhibitory concentration) and MBC (minimum bactericidal concentration)/MFC (minimum fungicidal concentration) were evaluated according to Bloomfield [22, 23]. By plotting natural logarithm of concentration (ln_C_) in *x*-axis versus zone of inhibition squared value (Z^2^) in *y*-axis, the value that crosses *x* = 0 would be Mt. Moreover, MIC was then calculated as 0.25 × Mt and MBC/MFC as 4 × MIC [23]. Mueller–Hinton agar was bored with a cork borer to make triplicate wells at equidistance aseptically and then inoculated with the prepared test microorganism (0.5 McFarland) aseptically. The volume (100 *µ*l) of each crude extract and fraction prepared at a twofold dilution was micropipetted into the wells, and the bacteria were incubated at 37°C and fungal at room temperature. Ceftriaxone, terbinafine, and dimethyl sulfoxide (DMSO) were used as positive, positive, and negative controls, respectively. Zones of inhibitions were expressed in mm according to clinical laboratory standards. All the experiments were done in triplicate to ensure representative data.

#### 2.4.4. Serial Microdilution Method

A serial microdilution assay as a microbial growth indicator was used to evaluate the minimum inhibitory concentrations (MICs) as described by Ramadwa et al. [24–26]. Twofold serial dilutions were placed into test tubes as follows: using an auto-dispenser, 2 ml of Mueller–Hinton Broth was dispensed in each test tube, 2 ml (500 mg/ml) of crude extract or its fraction was pipetted in each of the first tubes of the relevant series of dilutions, and thus the extract/fraction was diluted to half; 2 ml was removed from the first well and dispensed into the next tube on the series. The procedure was repeated on the series to the last test tube, and 2 ml from the last tube was discarded to ensure uniformity of volumes of extract/fraction. To each test tube, 100 *μ*l of subcultured standard microbes was micropipetted. The bacterial test tubes were incubated overnight at 37°C in a humidified oven and room temperature for fungus. The MIC was documented as the highest dilution or lowest concentration of the crude extract/fraction that inhibited antimicrobial growth assessed on turbidity. However, for the test tubes that showed no visible bacterial growth, pour plate method was used to determine MBC-least concentration at which no growth is visible after subculturing into a fresh broth. Ceftriaxone, terbinafine, and DMSO were used as positive, positive, and negative indicators, respectively. All the experiments were conducted in triplicate to ensure representative data.

### 2.5. Cytotoxicity Assay

#### 2.5.1. Brine Shrimp Hatching

*Artemia salina* eggs (Dohse Aquaristik GmbH & Co. KG, Germany) were incubated in a shallow plastic container with a partition and numerous 2 mm holes making two unequal sections. The plate was filled with 3.3% artificial saline solution which was prepared with marine/brine salt (Sera, Germany) and 50 mg yeast spread into the darkened section. The minor partition was lit by a tube light and moderately sparged with air. After 24–36 h, the shrimp larvae were moved to fresh artificial marine water and nurtured for additional 24 h under tube light. The shrimp larvae were collected via pipette from the illuminated compartment.

#### 2.5.2. Brine Shrimp Cytotoxicity Assay

Brine shrimp model as descried by Meyer et al. [27] was used to assay the lethality of the crude extracts and fractions of *Ocimum americanum* L. The crude extracts and fractions were prepared by weighing 50 mg, dissolved in 1 ml of 1% DMSO, and making up to 5 ml with brine solution. Ten shrimp larvae were macroscopically counted in the stem of a Pasteur pipette against an illuminated background; then transferred into sample tubes holding aliquots of 10 *µ*g/ml, 100 *µ*g/ml, and 1000 *µ*g/ml; and were made to 5 ml with marine solution. Vincristine and DMSO were used as positive and negative control, respectively. All the tubes were kept under artificial lighting. The dead shrimp larvae were counted with the help of a ×3 magnifying glass after 24 h. The mortality ratio in percentage at the trio dosage and control were calculated as % mortality = [(number of dead nauplii/total number of nauplii) × 100]. The surviving shrimp larvae were euthanized by addition of 100 *μ*l of phenol (5%) to each tube. All the experiments were carried out in quintuplicate.

### 2.6. Phytochemical Screening

The plant materials (extracts and fractions) of *Ocimum americanum* L. were qualitatively assayed for the presence of phytoconstituents such as alkaloids, anthraquinones, cyanogenic glycosides, cardiac glycosides, flavonoids, reducing sugars, tannins, saponins, terpenoids, phenolics, polyuronides, and phytosterols/triterpenes according to the techniques described by Evans, Harborne, and Pengelly [28–30].

### 2.7. Data Analysis

The data obtained in triplicate was cleaned and inputted into SPSS version 23. Using the software, descriptive statistics such as mean, standard error of the mean, and minimum and maximum values were determined. Means were used to generate graphs in Microsoft Excel. Probit analysis and linear regression were used to determine the median lethal concentration (LC_50_). Data was also subjected to inferential statistics where microbial growth inhibitions due to the various concentrations of the crude extract were compared to determine whether there were significant differences in growth inhibitions between the concentrations using one-way ANOVA. Post hoc Tukey HSD test was done to compare two growth inhibitions means at one go to determine whether they are significant or not, while post hoc Dunnett T test was also done to compare growth inhibition of each concentration with the growth inhibition observed in the controls. *P* value (significance level/probability level) set during the ANOVA and post hoc ANOVA analysis was 0.05.

## 3. Results

### 3.1. Percentage Yield of Crude Extracts

The acetonic, hydroethanolic, and aqueous crude extracts had yields of 1.154%, 2.6%, and 4.5%, respectively.

### 3.2. Phytochemical Profile

In the present study, the following phytoconstituents were present in the crude extracts and fractions of *Ocimum americanum* L.: alkaloids, anthraquinones, cardiac glycosides, cyanogenic glycosides, flavonoids, reducing sugars, saponins, tannins, terpenoids, phenolics, and phytosterols/triterpenes, as shown in Table 1. From the results, cardiac glycosides, flavonoids, reducing sugars, tannins, and phenolics were detected in all the samples while polyuronides were absent in all the samples.

### 3.3. Brine Shrimp Lethality

**Figure 1:**
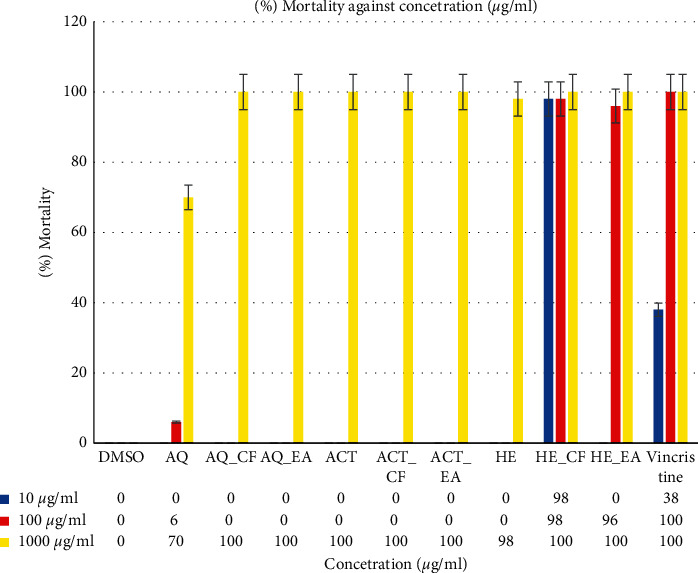
Brine shrimp % mortality against various concentrations of *O. americanum* L. crude extracts and their fractions.

The cytotoxicity results of crude extracts and their fractions of *Ocimum americanum* L. against the nauplii of *Artemia salina* are presented in Figure 1 and Table 2. On the Meyer's toxicity scale, LC_50_ of <1000 *µ*g/ml is toxic while >1000 *µ*g/ml is nontoxic [27, 31]. The LC_50_ values were construed by the use of Clarkson's scale of toxicity. The LC_50_ values >1000 *µ*g/ml were considered nontoxic, values between 501 and 1000 *µ*g/ml slightly cytotoxic, values of 101–500 *µ*g/ml moderately cytotoxic, and values within 0–100 *µ*g/ml highly cytotoxic [31, 32]. All samples were toxic with a 95% confidence interval ranging from LC_50_ 0.59 *µ*g/ml (chloroform fraction of the hydroethanolic extract) to LC_50_ 559.71 *µ*g/ml for aqueous sample. The chloroformic fraction of the hydroethanolic crude extract was more lethal than the positive control, vincristine with LC_50_ of 11.83 *µ*g/ml.

### 3.4. Antimicrobial Activity

The antimicrobial activity of various crude extracts and fractions of *Ocimum americanum* L. was evaluated using agar well diffusion and microdilution methods against opportunistic pathogens. The results on Tables 3 and 4 shows that all the extracts and fractions of acetone and hydroethanolic samples have good activity, while the aqueous crude extract and its fractions did not show any activity against the tested microbes.

Inhibition means which are statistically significant (*p* < 0.05) have been flagged with a different superscript letter while inhibition means which are not significant (*p* > 0.05) have been flagged with the same superscript letter. For instance in Table 3, *S. aureus* growth inhibition was not different between the three concentrations of 500 mg/ml, 250 mg/ml, and 125 mg/ml of acetone extract. These three inhibitions means are significantly different (*p* < 0.05) from the four smaller growth inhibition means from 62.5 mg/ml, 31.25 mg/ml, 15.62 mg/ml, and 7.81 mg/ml of acetone extract which are very close to each other and also are not significantly different when compared to each other. All the inhibition means from various concentrations of acetone extract were significantly different from the positive control inhibition.

Table 4 presents the MIC and MBC/MFC which were evaluated according to Bloomfield. This table compares the two methods by transforming the zones of inhibition into MIC and MBC and computing the ratio of MBC/MIC. Compounds/extracts that demonstrate antimicrobial properties are categorized as bactericidal when MBC/MIC ratio ≤4 and bacteriostatic when the ratio of MBC/MIC >4. Using this as a standard, we propose that acetone (MBC/MIC = 4.01) crude extract is bacteriostatic in nature while its fractions are bactericidal against *B. cereus*, *S. aureus*, and *C. albicans* microbes. The MIC for microdilution is indicated as ND (not determined) because all the samples were colored and thus turbidity was not visible; therefore, pour plate method was used to determine the MBC. This is a limitation of this particular method. On the agar well method, the Gram-negative bacteria were IE (ineffective), and the hydroethanolic crude extract and its fractions were ineffective for the fungus C*andida albicans.*

## 4. Discussion

Antimicrobial drug resistance (AMDR) has emerged as a result of indiscriminate use of antibiotic feed additives in animal husbandry and intensified by noncompliance of patients to the prescribed antimicrobial dosage regimens [1, 2]. Consequently, alternative strategies are needed to fight antimicrobial resistance, and the plant kingdom is the natural reservoir at our disposal [4]. Many studies have observed that plant extracts have good anti-infective, antioxidant, and anti-inflammatory activities due to their secondary metabolites [33]. Additionally, various species of *Ocimum* (basil) have been reported to show antimicrobial [4, 18], antifungal [17], anthelminthic [34], larvicidal [19, 35], nematocidal [36], and gastric cytoprotective antiulcer activities [15]. The results obtained from the current study show that the crude extracts and fractions of *Ocimum americanum* L. comprise numerous classes of secondary metabolites (Table 1) which are believed to be responsible for the observed ethnomedicinal properties [37]. The data have also indicated the effect of solvent choices on the extraction yield of the secondary metabolites from the herbal plant. The acetonic crude extract had the lowest percentage yield of 1.2%, followed by hydroethanolic crude extract which gave 2.6%, and the highest percentage yield was given by the aqueous crude extract at 4.5%.

All the crude extracts and their fractions demonstrated effective antimicrobial activities against the Gram-positive bacteria *B. cereus* and *S. aureus* and the fungus *C. albicans* but did not demonstrate any effects on Gram-negative (*E. coli* and *K. pneumoniae*) bacteria. However, the aqueous extracts and their fractions showed no antimicrobial activities, which has been reported by an earlier study [4]. Despite Gram-positive bacteria having thicker cell wall, their peptidoglycan layer is more permeable to certain compounds than the Gram-negative bacteria, due to the absence of the outer (lipopolysaccharide) membrane [4, 38]. Similarly, the inactivity of the aqueous crude extracts and their fractions is due to polarity; the nonpolar outer (lipopolysaccharide) membrane and phospholipid bilayer form a hydrophobic barrier, thus preventing entry of polar compounds inside the cell [39]. The ethyl acetate fraction of hydroethanolic crude extract exhibited highest inhibition zone at 250 mg/ml and 500 mg/ml against *B. cereus*, being 26.00 ± 0.00 and 26.50 ± 0.29, respectively, and not significantly different (*p* > 0.05) from the inhibition of ceftriaxone (30.00 ± 1.16), followed by the chloroform (18.17 ± 0.60) fraction of hydroethanolic crude extract (Table 2). Prospective antimicrobial hits are predicted to be liable on their zone of inhibition achieved from agar well diffusion technique [40]. In the current study, the results from agar well diffusion method confirmed that ethyl acetate fraction of hydroethanolic crude extract demonstrated strong antimicrobial activity. This has been confirmed by previous studies where ethyl acetate extract showed strong inhibition against *B. cereus*, as well as being highly toxic against brine shrimps larvae [4, 37].

Minimum inhibitory concentration (MIC) of crude extracts and fractions of *Ocimum americanum* L. was sensitive to *B. cereus*, *S. aureus*, *E.coli*, *K. pneumoniae*, and *C. albicans* but not sensitive to any of the concentrations of aqueous extracts and their fractions. The Gram-positive bacteria *B. cereus* and *S. aureus* were sensitive to all the crude extracts and fractions of acetone and hydroethanol (Table 4), while the fungus *C. albicans* was only sensitive to the crude extracts and fractions of acetone. Minimum bactericidal/fungicidal concentration (MBC/MFC) of the crude extracts and fractions of *Ocimum americanum* L. was sensitive to all microbes except the crude extract and fractions while hydroethanolic crude extracts and fractions were not sensitive to the fungus *C. albicans* (Table 4). Compounds/extracts that demonstrate antimicrobial properties are categorized as bactericidal when MBC/MIC ratio ≤4 and bacteriostatic when the ratio of MBC/MIC >4 [41, 42]. Using this as a standard, we propose that acetone (MBC/MIC = 4.01) crude extract is bacteriostatic in nature while its fractions are bactericidal against *B. cereus*, *S. aureus*, and *C. albicans* microbes. Similarly, we noted that hydroethanolic crude extract and fractions are bactericidal against Gram-positive bacteria but not fungus. Nevertheless, discretion ought to be exercised in interpretation of the data, since the higher the MIC values, the more probable that the analyzed data lose clinical significance [41]. Additionally, various studies have stated much lower MIC values against Gram-positive bacteria. We postulate that the variances in MIC data reported between our results and those of other studies may be due to the variations in the presence of secondary metabolites among the same species of the plant [4, 37]. The current study demonstrated lower antimicrobial activity when compared to the positive controls (ceftriaxone and terbinafine) at the equal concentration of 250 mg/ml. It was demonstrated that there is no significant variation (*p* > 0.05) in the mean zones of inhibition of the crude extracts and fractions of acetone and hydroethanol. On the contrary, there was a statistical significance (*p* < 0.05) between the mean zones of inhibition for the two crude extracts and their fractions compared to the positive controls under various concentrations.

The toxicological assay constituted by *Artemia salina* nauplii is generally used to evaluate different physiological and toxicological properties of herbal crude extracts and is regarded as prognostic of cytotoxicity [37]. The model demonstrated a potential cytotoxicity (low LC_50_ values) of all crude extracts and fractions of *Ocimum americanum* L. (Table 2). The analyzed results gave very low LC_50_ on Clarkson's scale [31, 32]. Chloroform and ethyl acetate fractions of hydroethanolic crude extract demonstrated the highest toxicological profile against brine shrimp larvae, and this is reported for the first time. The chloroform (LC_50_ 0.59 *µ*g/ml) fraction had lower median lethal concentration compared to the standard drug vincristine (LC_50_ 11.83 *µ*g/ml), which shows a high potential for chloroformic fraction as novel compound and that further bioprospecting should be considered. Similarly, the ethyl acetate (LC_50_ 44.65 *µ*g/ml) fraction was highly toxic compared to Clarkson's criteria. The aqueous crude extract was slightly toxic (LC_50_ 559.71 *µ*g/ml), and this explains the observed results that the herbal concoction/decoction for acute use does not show any signs or symptoms of toxicity in the patients who use them. The acetonic crude extract and fractions as well as the aqueous fractions had moderate toxicity (LC_50_ 303.39 *µ*g/ml), which shows that they have a low partition coefficient (Kd), which weakens the extraction and partitioning power of these solvents [43]. The disparity in the toxicity between the crude extracts and the fractions is due to the phytochemical distribution of alkaloids, flavonoids, phenols, tannins, and terpenoids on the samples [38, 44].

The phytochemical screening in the current study reports the presence of several secondary metabolites. These include alkaloids, anthraquinones, cardiac glycosides, cyanogenic glycosides, flavonoids, reducing sugars, saponins, tannins, terpenoids, phenolics, and phytosterols/triterpenes. Polyuronides were not detected in any of the samples (Table 1). The alkaloids were present in aqueous crude extracts and their fractions and hydroethanolic crude extract but not in the acetone crude extract and its fractions. Alkaloids have biological properties in faunae [45]. They impede the cyclooxygenase cascade which in turn prevents interleukins and cytokines that initiate pain during inflammatory process. Research has also demonstrated that alkaloids possess antimicrobial, antimalarial, and antispasmodic activities [38, 45]. The anthraquinones were only present in the acetone extract and its chloroform fraction. The cardiac glycosides were present in all the samples. Commonly found as secondary metabolites in various plants, these phytocompounds have a varying array of biophysiological effects on myocardia function and have also been proposed for use in tumor chemotherapy [46]. Cyanogenic glycosides were only present in aqueous and hydroethanolic extract as well as in acetone and ethyl acetate fraction of acetone extract. The flavonoids, reducing sugars, tannins, and phenolics were present in all the samples. Among the secondary metabolites, flavonoids and phenolics compounds are the most active group of phytochemicals. These compounds have broad spectrum of pharmacological properties including antimicrobial activities [4]. Phenolics and flavonoids compounds inhibit peptidoglycan and nucleic acid synthesis due to their hydroxyl moiety, thus blocking the synthesis of bacterial cell wall [47, 48]. These findings are consistent with Zengin et al. who reported that the contents of flavonoids and phenols have inhibitory effects against certain bacteria and fungus strains [37, 49]. Tannins were present in all the crude extracts and factions of *Ocimum americanum* L. They are water-soluble polyphenolic biomolecules which are found in many medicinal plants. Sieniawska and Baj in a review observed that high content of tannins is beneficial due to their antimicrobial and antitumor properties, which is consistent with our findings [50, 51]. Bioactive saponins were present in hydroethanolic and aqueous crude extracts and their fractions but not in acetone crude extract and its fractions. The saponins are a subclass of terpenoids, typically bitter tasting, plant-derived toxic organic compounds that are frothy when shaken in water. Saponins have varying activities, both beneficial and toxic. Clinical studies have reported that saponins are cytotoxic due to induction of apoptosis and modulation of the immune system against rapid growing tumor cells [52]. The phytosterols/triterpenes were present in the nonaqueous solvents, i.e., were present in the acetone extract and all the fractions. Phytosterols related to cholesterol are structural components of plant physiological membranes. Phytosterols are chemopreventive compounds and are reported to have antitumor properties through various mechanisms [53]. McCann et al. observed that dietary phytosterol intake reduces the risk of several types of cancers including ovarian cancer [54, 55].

## 5. Conclusion

This study seeks to demonstrate the activity and ensure the efficacy and safety of ethnobotanically utilized *Ocimum americanum* L. The study explored the antimicrobial activity, cytotoxicity, and phytochemical composition of *Ocimum americanum* L. crude extracts and fractions. The assay data from the current research prove that the herbal medicinal plant *Ocimum americanum* L. contains several secondary metabolites which are responsible for the antimicrobial activity and cytotoxic effects. The results also feature the effect of solvent choice on the extraction yield of the crude extracts and fractions from the plant. The chloroformic fraction of hydroethanolic crude extract showed high cytotoxicity and is thus a potential candidate for bioprospecting. Ethyl acetate fraction of hydroethanolic extract has shown potential antimicrobial activity against Gram-positive bacteria, in particular *B. cereus*. Chloroformic and ethyl acetate fractions of hydroethanol crude extract possess prospective compounds which are responsible for cytotoxic and antimicrobic activities, respectively. Findings from the current study will serve as a potential basis for discovery of a novel bioactive natural product. Subsequently, more research is required for compounds separation and isolation, chronic toxicity, and clinical studies of the active biomolecules.

## Figures and Tables

**Table 1:** Phytochemical profile of *Ocimum americanum* L. crude extracts and their fractions.

Phytochemical screening									
Phytochemical	AQ	AQ_CF	AQ_EA	ACE	ACE_CF	ACE_EA	HE	HE_CF	HE_EA
Alkaloids	+	+	+	−	−	−	+	−	−
Anthraquinones	−	−	−	+	+	−	−	−	−
Cardiac glycosides	+	+	+	+	+	+	+	+	+
Cyanogenic glycosides	+	−	−	+	−	+	+	−	−
Flavonoids	+	+	+	+	+	+	+	+	+
Reducing sugars	+	+	+	+	+	+	+	+	+
Saponins	+	+	−	−	−	−	+	+	+
Tannins	+	+	+	+	+	+	+	+	+
Terpenoids	−	+	+	+	+	+	−	+	+
Phenolics	+	+	+	+	+	+	+	+	+
Polyuronides	−	−	−	−	−	−	−	−	−
Phytosterols	−	+	+	+	+	+	−	+	+

*Note*. (+): presence of phytochemical; (−): absence of phytochemical. AQ: aqueous crude extract; ACE: acetone crude extract; HE: hydroethanolic crude extract; AQ_CF: chloroform fraction of the aqueous crude extract; AQ_EA: ethyl acetate fraction of the aqueous crude extract; ACE_CF: chloroform fraction of the acetone crude extract; AC_EA: ethyl acetate fraction of the acetone crude extract; HE_CF: chloroform fraction of the hydroethanolic crude extract; HE_EA: ethyl acetate fraction of the hydroethanolic crude extract.

**Table 2:** Summary of mortality per test dose of *Ocimum americanum* L. crude extracts and fractions against brine shrimp larvae (*Artemia salina* nauplii).

Sample	Mortality per test dose			LC_50_ *µ*g/mL	95% CI	Clarkson's scale
	10 *µ*g/mL	100 *µ*g/mL	1000 *µ*g/mL			
Vincristine: positive control	19	50	50	11.83	–	Highly toxic
Acetone crude extract	0	0	50	303.39^a^	198.42–488.86	Moderately toxic
Chloroform fraction of the acetone extract	0	0	50	303.39^a^	198.42–488.86	Moderately toxic
Ethyl acetate fraction of the acetone extract	0	0	50	303.39^a^	198.42–488.86	Moderately toxic
Aqueous crude extract	0	3	35	559.71	397.09–811.09	Slightly toxic
Chloroform fraction of the aqueous extract	0	0	50	303.39^b^	198.42–488.86	Moderately toxic
Ethyl acetate fraction of the aqueous extract	0	0	50	303.39^b^	198.42–488.86	Moderately toxic
Hydroethanolic crude extract	0	0	49	347.80	217.06–498.10	Moderately toxic
Chloroform fraction of the hydroethanolic crude extract	49	50	50	0.59	–	Highly toxic
Ethyl acetate fraction of the hydroethanolic crude extract	0	48	50	44.65	4.65–65.86	Highly toxic

The extracts and fractions denoted by _a_ and _b_ have no significant difference (*p* > 0.05) in between the groups and in the same group.

**Table 3:** Inhibition of microbial growth by *Ocimum americanum* L. crude extracts and their fractions on twofold serial dilutions using the agar well diffusion method.

Microorganism	Conc mg/ml	Zone of inhibition (MM)						Positive control	Negative control
		Acetone extract	Chloroform fraction	Ethyl acetate fraction	Hydroethanol extract	Chloroform fraction	Ethyl acetate fraction		
*Bacillus cereus* (gram +ve)	500	15.67^b^ ± 0.33	15.50^b^ ± 0.50	14.00^b^ ± 0.58	16.33^b^ ± 0.67	20.00^b^ ± 0.58	26.50^ab^ ± 0.29	30.00^a^ ± 1.16	0.00^d^ ± 0.00
	250	14.33^b^ ± 0.67	14.33^b^ ± 0.33	13.00^b^ ± 0.58	13.17^c^ ± 0.17	18.17^b^ ± 0.60	26.00^ab^ ± 0.00		
	125	13.33^b^ ± 0.33	13.50^b^ ± 0.29	13.00^b^ ± 0.00	10.83^d^ ± 0.44	14.83^c^ ± 0.44	23.83^c^ ± 0.60		
	62.5	13.83^b^ ± 0.17	12.00^c^ ± 0.58	11.50^c^ ± 0.29	11.00^d^ ± 0.00	9.83^c^ ± 4.94	20.00^d^ ± 0.58		
	31.25	12.00^c^ ± 0.58	11.67^c^ ± 0.33	11.83^c^ ± 0.17	9.83^d^ ± 0.44	13.00^c^ ± 0.58	17.50^e^ ± 0.29		
	15.62	11.50^c^ ± 0.29	11.33^c^ ± 0.33	10.67^c^ ± 0.17	9.67^d^ ± 0.33	9.33^c^ ± 0.33	14.33^f^ ± 0.44		
	7.81	10.67^c^ ± 0.33	9.50^e^ ± 0.29	10.00^c^ ± 0.29	9.50^d^ ± 0.29	0.00^g^ ± 0.00	13.17^f^ ± 0.17		

*Escherichia coli*	500	0.00^b^ ± 0.00	0.00^b^ ± 0.00	0.00^b^ ± 0.00	0.00 ± 0.00	0.00 ± 0.00	0.00 ± 0.00	45.00^a^ ± 0.58	0.00^d^ ± 0.00

*Staphylococcus aureus* (gram +ve)	500	14.50^b^ ± 0.29	12.00^b^ ± 0.58	11.17^b^ ± 0.44	17.17^b^ ± 0.44	18.50^b^ ± 0.29	16.83^b^ ± 0.60	50.00^a^ ± 1.15	0.00^d^ ± 0.00
	250	14.66^b^ ± 0.33	10.67^b^ ± 0.33	10.00^b^ ± 0.29	13.33^c^ ± 0.33	15.67^c^ ± 0.44	16.17^b^ ± 0.17		
	125	14.00^b^ ± 0.58	9.67^c^ ± 0.33	9.17^b^ ± 0.17	10.50^d^ ± 0.29	12.83^d^ ± 0.44	11.83^b^ ± 0.17		
	62.5	12.00^c^ ± 0.58	9.33^c^ ± 0.33	9.33^b^ ± 0.33	9.83^d^ ± 0.17	9.67^e^ ± 0.17	9.83^c^ ± 0.44		
	31.25	11.66^c^ ± 0.88	9.67^c^ ± 0.33	0.00^d^ ± 0.00	6.17^d^ ± 3.09	9.17^e^ ± 0.17	6.17^c^ ± 3.09		
	15.62	11.00^c^ ± 0.00	9.00^c^ ± 0.00	0.00^d^ ± 0.00	3.00^e^ ± 3.00	6.00^e^ ± 3.00	0.00^f^ ± 0.00		
	7.81	9.67^c^ ± 0.33	0.00^d^ ± 0.00	0.00^d^ ± 0.00	3.00^e^ ± 3.00	0.00^f^ ± 0.00	0.00^f^ ± 0.00		

*Klebsiella pneumoniae*	500	0.00^b^ ± 0.00	0.00^b^ ± 0.00	0.00^b^ ± 0.00	0.00^b^ ± 0.00	0.00^b^ ± 0.00	0.00^b^ ± 0.00	50.00^a^ ± 1.73	0.00^b^ ± 0.00

*Candida albicans* (fungus)	500	15.00^b^ ± 0.58	16.83^b^ ± 0.17	15.50^b^ ± 0.29	0.00^b^ ± 0.00	0.00^b^ ± 0.00	0.00^b^ ± 0.00	45.00^a^ ± 1.73	0.00^d^ ± 0.00
	250	14.33^b^ ± 0.33	14.67^c^ ± 0.33	9.00^b^ ± 4.51	0.00^b^ ± 0.00	0.00^b^ ± 0.00	0.00^b^ ± 0.00		
	125	12.33^c^ ± 0.33	12.00^d^ ± 0.58	12.50^b^ ± 0.29	0.00^b^ ± 0.00	0.00^b^ ± 0.00	0.00^b^ ± 0.00		
	62.5	11.17 ± 0.17	10.00^e^ ± 0.58	10.67^b^ ± 0.58	0.00^b^ ± 0.00	0.00^b^ ± 0.00	0.00^b^ ± 0.00		
	31.25	9.83^d^ ± 0.44	9.17^e^ ± 0.27	6.33^b^ ± 3.18	0.00^b^ ± 0.00	0.00^b^ ± 0.00	0.00^b^ ± 0.00		
	15.62	0.00^e^ ± 0.00	0.00^g^ ± 0.00	3.17^c^ ± 3.17	0.00^b^ ± 0.00	0.00^b^ ± 0.00	0.00^b^ ± 0.00		
	7.81	0.00^e^ ± 0.00	0.00^g^ ± 0.00	0.00^c^ ± 0.00	0.00^b^ ± 0.00	0.00^b^ ± 0.00	0.00^b^ ± 0.00		

Means flagged with the same superscript letter within the considered group, i.e., zone of inhibition, are not significantly different (*p* > 0.05). Positive control mean is flagged with “a”; negative control with “d”; and 500, 250, 125 mg/ml with “b” implying they are statistically the same/they had the same activity against *S. aureus*; and 62.5, 31.25, 15.62, and 7.81 mg/ml with “c,” because they also had statistically the same inhibitory activity against *S. aureus*; i.e., their differences are statistically significant (*p* < 0.05).

**Table 4:** MIC, MBC, and MFC of *Ocimum americanum* L. crude extracts and their fractions of various solvents against test microbes.

Pathogen		Macro dilution (mg/mL)			Agar well diffusion (mg/mL)		
	Sample	MIC (mg/mL)	MBC (mg/mL)	MBC/MIC	MIC	MBC	MBC/MIC
*Bacillus cereus* (gram +ve)	**Acetone crude extract**	ND	208.33 ± 72.17	—	1.93 ± 0.14	7.74 ± 0.55	4.01
	Chloroform fraction	ND	166.67 ± 72.17	—	1.38 ± 0.34	5.50 ± 1.37	3.99
	Ethyl acetate fraction	ND	166.67 ± 72.17	—	1.91 ± 0.15	7.64 ± 0.61	4.00
	**70% hydroethanolic crude extract**	ND	208.33 ± 72.17	—	1.54 ± 0.09	6.15 ± 0.37	3.99
	Chloroform fraction	ND	83.33 ± 36.08	—	2.70 ± 0.81	10.81 ± 3.26	4.00
	Ethyl acetate fraction	ND	166.67 ± 72.17		3.43 ± 0.23	13.71 ± 0.93	4.00

*Staphylococcus aureus* (gram +ve)	**Acetone crude extract**	ND	52.08 ± 18.04	—	1.49 ± 0.62	5.95 ± 2.50	3.99
	Chloroform fraction	ND	104.17 ± 36.08	—	0.72 ± 0.53	2.90 ± 2.11	4.03
	Ethyl acetate fraction	ND	166.67 ± 72.17	—	2.26 ± 0.06	9.01 ± 0.22	3.99
	**70% hydroethanolic crude extract**	ND	208.33 ± 72.17	—	1.84 ± 0.78	7.34 ± 3.12	3.99
	Chloroform fraction	ND	83.33 ± 36.08	—	2.87 ± 0.21	11.46 ± 0.84	3.99
	Ethyl acetate fraction	ND	104.17 ± 36.08		3.34 ± 0.14	13.37 ± 0.55	4.00

*Escherichia coli* (gram −ve)	**Acetone crude extract**	ND	166.67 ± 72.17	—	IE	IE	—
	Chloroform fraction	ND	83.33 ± 36.08	—	IE	IE	—
	Ethyl acetate fraction	ND	166.67 ± 72.17	—	IE	IE	—
	**70% hydroethanolic crude extract**	ND	83.33 ± 36.08	—	IE	IE	—
	Chloroform fraction	ND	20.83 ± 9.02	—	IE	IE	—
	Ethyl acetate fraction	ND	41.67 ± 18.04		IE	IE	—

*Klebsiella pneumoniae* (gram −ve)	**Acetone crude extract**	ND	145.83 ± 95.47	—	IE	IE	—
	Chloroform fraction	ND	250.00 ± 0.00	—	IE	IE	—
	Ethyl acetate fraction	ND	83.33 ± 36.08	—	IE	IE	—
	**70% hydroethanolic crude extract**	ND	333.33 ± 144.33	—	IE	IE	—
	Chloroform fraction	ND	208.33 ± 72.17	—	IE	IE	—
	Ethyl acetate fraction	ND	208.33 ± 72.17	—	IE	IE	—

*Candida albicans* (Fungi)	**Acetone crude extract**	ND	250.00 ± 0.00	—	2.87 ± 0.18	11.46 ± 0.74	3.99
	Chloroform fraction	ND	166.67 ± 72.17	—	2.88 ± 0.31	11.52 ± 1.24	4.00
	Ethyl acetate fraction	ND	166.67 ± 72.17	—	2.65 ± 0.58	10.58 ± 2.30	3.99
	**70% hydroethanolic crude extract**	ND	IE	—	IE	IE	—
	Chloroform fraction	ND	IE	—	IE	IE	—
	Ethyl acetate fraction	ND	IE		IE	IE	—

MIC: minimum inhibitory concentration; MBC: minimum bactericidal concentration; MBC/MIC: ratio of minimum inhibitory concentration to minimum bactericidal concentration; ND: not determined; IE: ineffective.

## Data Availability

The results presented to demonstrate the findings of the current study are accessible from the corresponding author upon request.
